# Association of Cognitive Impairment With Anhedonia in Patients With Schizophrenia

**DOI:** 10.3389/fpsyt.2021.762216

**Published:** 2021-12-02

**Authors:** Lingfang Yu, Hua Ni, Zenan Wu, Xinyu Fang, Yan Chen, Dandan Wang, Chen Zhang

**Affiliations:** ^1^Schizophrenia Program, Shanghai Mental Health Center, Shanghai Jiao Tong University School of Medicine, Shanghai, China; ^2^Shanghai Xuhui Mental Health Center, Shanghai, China; ^3^The Affiliated Brain Hospital of Nanjing Medical University, Nanjing, China

**Keywords:** schizophrenia, anticipatory anhedonia, consummatory anhedonia, cognitive function, language, delayed memory

## Abstract

Anhedonia is considered as one of the five dimensions of negative symptoms and mainly refers to the reduction of the capacity of feeling pleasure. Increasing evidence suggests that anhedonia in schizophrenia may be partly explained by cognitive impairment. However, the associations between specific cognitive impairment and anhedonia are not fully investigated. The purpose of this study was to examine anticipatory anhedonia, consummatory anhedonia, and their cognitive associations in schizophrenia. A total number of 100 patients with schizophrenia and 67 healthy volunteers were recruited. The clinical symptoms of schizophrenia were assessed. Anticipatory pleasure, consummatory pleasure, and cognitive functions of each participant were measured. Multiple linear regression analysis was performed to investigate the influencing factors of anhedonia in schizophrenia. The results showed no significant differences in sex, age, education year, body mass index (BMI), and marital status between the schizophrenia group and healthy control group (all *P* > 0.05). Both anticipatory and consummatory pleasure in the schizophrenia group were significantly lower than those in the healthy control group (all *P* < 0.05). Immediate memory, visual spanning, language, attention, and delayed memory were significantly poorer in the schizophrenia group (all *P* < 0.05). The results showed that language deficit is an independent risk factor for anticipatory anhedonia (*B'* = 0.265, *P* = 0.008, 95% *CI:* 0.038-0.244), while delayed memory deficit is an independent risk factor for consummatory anhedonia (*B'* = 0.391, *P* < 0.001, 95% *CI:*0.085-0.237). To the best of our knowledge, this is the first study that reported the specific cognitive associations of anhedonia in schizophrenia. The findings have added new evidence on the influencing factors of anhedonia and provided clues for the associations between clinical manifestations of schizophrenia.

## Introduction

Schizophrenia is a severe mental illness with diversified and complex symptoms. It affects ~1% of the population and has caused a substantial healthcare burden (1). Patients with schizophrenia are suggested to receive long-term treatment since schizophrenia is a disorder that easily relapses. However, residual symptoms such as negative symptoms and cognitive impairment persist long after treatment and have a significant impact on quality of life (2).

Anhedonia is a common symptom shared across psychiatric disorders such as schizophrenia, depression, and addiction (3, 4). It is mainly defined as the reduced capacity of experiencing the feeling of pleasure. It has been classified as one of the five dimensions of negative symptoms of schizophrenia according to the Measurement and Treatment Research to Improve Cognition in Schizophrenia (MATRICS). So far anhedonia has little response to antipsychotic treatments. And accumulating evidence has proven that anhedonia is closely related to poor outcomes in schizophrenia, including a higher risk of suicidal ideation and poorer functional recovering (5–7). Therefore, it is of great importance to clarify the features and influencing factors of anhedonia in schizophrenia.

The findings of existing literature focusing on anhedonia in schizophrenia are inconsistent. Some studies using self-rating scales show that patients with schizophrenia has a high level of anhedonia (8–10). While other studies report that patients with schizophrenia share the same emotional response as healthy volunteers (11–13). More recently, anhedonia is divided into anticipatory pleasure and consummatory pleasure. The former refers to the reduced capacity of feeling pleasure derived from predicted future events. The latter refers to the reduced capacity of feeling pleasure during current events. These two dimensions of anhedonia may have different features and biological mechanisms. Although it is generally thought that anticipatory pleasure rather than consummatory pleasure is reduced in schizophrenia, literature has suggested inconsistent results (14–16).

Cognitive impairment is also a core aspect of schizophrenia. And cognitive function has been a potential explanation for anhedonia in psychosis (17). It requires complex cognitive skills such as drawing on past experiences, imagination reflection, and maintaining images to anticipate whether future events would bring the feeling of pleasure (18). People may have difficulties in bringing forth and holding images of positive event that can produce a feeling of pleasure due to the diminished working memory (18, 19). A study aiming at investigating social and physical anhedonia in patients with schizophrenia revealed the positive association between impaired verbal functions and elevated physical anhedonia (20), and a relationship between physical anhedonia and concept formation has been reported in non-affected first-degree relatives of schizophrenia patients (21). It is also reported that individuals with social anhedonia show poorer neurocognitive functions including attentional vigilance and simple processing speed (22). In a meta-analysis, the authors report that anticipatory anhedonia in people with psychosis is moderated by cognitive functions (23). There are evidence from neuroimage studies showing that language involved brain areas may also be involved in anhedonia (24). Moreover, semantic fluency, part of the language skills, is a proxy of initiation ability (25). And deficits in initiation ability can lead to difficulty in starting the prediction of future positive events. As for consummatory pleasure, it has been reported that in-the-moment feelings are impaired in schizophrenia patients with working memory deficits (26). Therefore, we hypothesized that language skills might be related to anticipatory pleasure, and delayed memory might be related to consummatory pleasure.

Although there are several lines of evidence giving a hint about the correlation between anhedonia and cognitive impairments, no study has investigated the distinctions in influencing cognitive factors for anticipatory anhedonia and consummatory anhedonia. To the best of our knowledge, this is the first study to report the specific associations between anhedonia (anticipatory and consummatory anhedonia) and domains of cognitive impairments in schizophrenia.

In the present study, we used the Repeatable Battery for the Assessment of Neuropsychological Status (RBANS) to assess the cognitive functions of schizophrenia, which contains the tests of immediate memory, visual spanning, language, attention, and delayed memory (27). The Temporal Experience of Pleasure (TEPS) was used to assess both anticipatory pleasure and consummatory pleasure (28). The purpose of this study was to examine anticipatory anhedonia and consummatory anhedonia and their cognitive associations in schizophrenia.

## Methods

### Participants

The schizophrenia patients were recruited from Shanghai Mental Health Center, and each subject was diagnosed by two independent psychiatrists. Healthy volunteers were recruited through advertisements in Shanghai and the surrounding areas. The inclusion criteria for schizophrenia patients were as follows: (1) met the criteria of schizophrenia in the *Diagnostic and Statistical Manual of Mental Disorders, Fourth Edition* (DSM-IV); (2) had a minimum education year of nine; (3) was aged from 18 to 50; (3) was Chinese Han; and (4) did not take antipsychotic medicine or any other psychotropic drugs within 2 weeks. And the patients were excluded if they: (1) had a diagnosis of other mental illness except for schizophrenia; (2) had severe somatic disease; and (3) were pregnant or lactating females. The inclusion criteria for healthy controls were as follows: (1) was aged from 18 to 50; (2) had a minimum education year of nine; and (3) was Chinese Han. The exclusion criteria for the healthy controls were as follows: (1) was patient with mental illness or close relative of patients with mental illness, or used any psychotropic drugs within 2 weeks; (2) had severe somatic diseases; and (3) were pregnant or lactating females. All participants were informed and signed the written informed consent before the initiation of the study.

### Measures

#### Demographic Information Collection

Participants' age, sex, education level, BMI, and marital status were recorded. In the schizophrenia group, the duration of illness and time since last antipsychotic use were also collected.

#### Clinical Assessment

The Positive and Negative Syndrome Scale (PANSS) was used for assessing psychiatric symptoms of schizophrenia, which can be divided into the PANSS positive, PANSS negative, and PANSS general psychopathology subscales (29). It is a widely used scale in schizophrenia research. TEPS was used for assessing anticipatory pleasure and consummatory pleasure of each participant. TEPS is composed of 20 items that can assess either anticipatory pleasure or consummatory pleasure. RBANS was performed to measure the cognitive functions of each subject. RBANS has the advantage of simple operation and it only takes 20-30 min to finish the test. Therefore, it is widely used and works well in cognitive assessment (27). Two psychiatrists who are well-trained for this project conducted the measurement. And the inter-rater correlation coefficient >0.8.

### Statistical Analysis

SPSS 26.0 software was used to perform the statistical analysis. Test for normality was performed using the *Kolmogorov–Smirnov* test. Between-group comparison of normally distributed data was performed using the independent sample *t*-test. Categorical data were tested using the chi-square test. Partial correlation analysis was performed to find the factors that related to anticipatory and consummatory pleasure, with age, sex, and education level as covariates. Finally, a stepwise multiple linear regression was conducted to explore the influencing cognitive factors of anticipatory and consummatory pleasure in schizophrenia, with age, sex, education level, and psychiatric symptoms as covariates. All statistical tests were two-tailed, and a value of *p* < 0.05 was considered significant.

## Results

### Demographic and Clinical Variables

A total of 100 patients with schizophrenia and 67 healthy volunteers were recruited. The age, education level, BMI, RBANS scores, and TEPS scores in each group were normally distributed (Kolmogorov–Smirnov one-sample test: all *P* > 0.05). No significant differences in age, sex, education level, BMI and marital status between the schizophrenia group and healthy control group were found. Compared to the healthy control group, the schizophrenia group had a lower level of both anticipatory and consummatory pleasure (*P* < 0.05). Besides, the schizophrenia group showed significantly decreased cognitive function (*P* < 0.05). Details are presented in Table 1.

**Table 1:** Demographic and clinical characteristics of participants.

	**Schizophrenia group (*n* = 100)**	**Healthy control group (*n* = 67)**	**t/X^**2**^**	***P***
Age/year	29.60 ± 8.300	28.43 ± 6.382	0.974	0.332
Sex			0.763	0.424
Male	38(38.00%)	30(44.78%)		
Female	62(62.00%)	37(55.22%)		
Education level/year	13.52 ± 2.894	14.39 ± 2.724	−1.945	0.054
Duration of illness/month	12.79 ± 17.335			
Time since last antipsychotic use /day	71.86 ± 84.700			
BMI /kg·m^−2^	21.95 ± 3.468	21.42 ± 2.95	1.040	0.300
**RBANS**				
Immediate memory	68.46 ± 18.513	92.18 ± 16.226	−8.520	<0.001
Visual spanning	86.12 ± 17.631	101.25 ± 15.322	−5.721	<0.001
Language	76.19 ± 16.646	96.15 ± 14.980	−7.901	<0.001
Attention	89.66 ± 18.415	114.22 ± 13.168	−9.420	<0.001
Delayed memory	71.94 ± 19.904	96.31 ± 12.985	−8.383	<0.001
Total score	72.70 ± 15.902	99.84 ± 13.988	−11.334	<0.001
**TEPS**				
Anticipatory pleasure	38.63 ± 8.851	46.48 ± 6.404	−6.242	<0.001
Consummatory pleasure	31.76 ± 8.181	39.54 ± 7.082	−6.348	<0.001
Total score	70.39 ± 16.123	86.01 ± 11.474	−6.852	<0.001
**PANSS**				
Positive symptom	27.22 ± 18.041			
Negative symptom	19.43 ± 8.097			
General psychopathology	41.50 ± 13.753			
Total score	80.35 ± 26.331			

*BMI, Body mass index; PANSS, Positive and Negative syndrome scale; RBANS, Repeatable Battery for the Assessment of Neuropsychological Status; TEPS, Temporal Experience of Pleasure Scale. Data were presented in Mean ± SD or N(%)*.

### Cognitive Associations With Anhedonia in Schizophrenia

Partial correlation analysis showed that anticipatory pleasure is associated with language (*r* = 0.265, *P* = 0.008) and delayed memory (*r* = 0.230, *P* = 0.022), while consummatory pleasure is associated with negative symptoms (*r* = −0.268, *P* = 0.007), general psychopathology (*r* = −0.243, *P* = 0.015), PANSS total score (*r* = 0.287, *P* = 0.015), immediate memory (*r* = 0.250, *P* = 0.012), language (*r* = 0.325, *P* = 0.001), attention (*r* = 0.218, *P* = 0.030) and delayed memory (*r* = 0.391, *P* = 0.000). Detailed information is presented in Table 2.

**Table 2:** Correlations between clinical variables and pleasure in the schizophrenia group.

**Pleasure**	**Clinical factors**	***r***	***P***
Anticipatory pleasure	Positive symptom	−0.118	0.241
	Negative symptom	−0.193	0.055
	General psychopathology	−1.131	0.195
	PANSS total score	−0.179	0.075
	Immediate memory	0.121	0.229
	Visual spanning	−0.025	0.808
	Language	0.265	0.008
	Attention	0.128	0.204
	Delayed memory	0.230	0.022
	RBANS total score	0.181	0.071
Consummatory pleasure	Positive symptom	−0.180	0.074
	Negative symptom	−0.268	0.007
	General psychopathology	−0.243	0.015
	PANSS total score	0.287	0.015
	Immediate memory	0.250	0.012
	Visual spanning	0.125	0.216
	Language	0.325	0.001
	Attention	0.218	0.030

*PANSS, Positive and Negative syndrome scale; RBANS, Repeatable Battery for the Assessment of Neuropsychological Status; TEPS, Temporal Experience of Pleasure Scale*.

Regression analysis showed that language is an independent risk factor of anticipatory pleasure (*B'*=0.265, *P* = 0.008, 95% *CI:* 0.038-0.244), and delayed memory is an independent risk factor of consummatory pleasure (*B'* = 0.391, *P* = 0.000, 95% *CI*: 0.085-0.237). Detailed information is presented in Table 3.

**Table 3:** Results of the stepwise multiple linear stepwise regression.

**Pleasure**	**Clinical factors**	***B***	***SE***	***B'***	***t***	***P***	**95%** ***CI*** **for** ***B***	
							**lower**	**upper**
Anticipatory pleasure	Language	0.141	0.052	0.265	2.721	0.008	0.038	0.244
Consummatory pleasure	Delayed memory	0.161	0.038	0.391	4.206	0.000	0.085	0.237

*B, regression coefficient; SE, standard error; B', standardized regression coefficient; CI, confidence interval*.

## Discussion

The present study showed that both anticipatory and consummatory pleasure were significantly decreased in patients with schizophrenia compared to healthy controls. This finding was in line with the results of some previous research (30). However, it should be mentioned that many studies are showing different results. The possible reasons for the conflicting results could be as follows. First, the heterogeneity of schizophrenia itself might be an important reason. The patients recruited could vary greatly among researches. Therefore, patients might exhibit great differences in clinical manifestations. Second, most antipsychotic drugs exert effects through the impact on dopamine or serotonin receptors in brain, which could influence clinical manifestations. In the present study, patients who did not take antipsychotic drugs within 2 weeks were recruited. However, many studies recruited schizophrenia patients who take antipsychotic drugs regularly. Hence the results might be different. Third, schizophrenia usually has a long illness duration, and the disease characteristics differ greatly in different stages of the disease. For example, the study of Mote recruited patients with short disease duration (<1 year), while other studies did not restrict disease duration (14). This might also influence the results.

In the present study, patients with schizophrenia showed significantly decreased global cognitive function compared to healthy controls, which was in line with our previous study (31). The associations between anhedonia and cognitive factors in schizophrenia were also confirmed in our study. Specifically, language deficit is a risk factor for anticipatory anhedonia, while delayed memory deficit is a risk factor for consummatory anhedonia. This accords with the common knowledge of the cognitive influence on anhedonia in schizophrenia. More importantly, we further assure the specific cognitive factors that may influence anticipatory and consummatory anhedonia for the first time.

Similar to our findings, impaired verbal function is reported to correlate with physical anhedonia in schizophrenia (20). The language test conducted in the present study contains picture naming and semantic fluency. Semantic fluency is a proxy of initiation ability (25), and the reduced ability of initiation may represent one's avolition. Anhedonia and avolition are both negative symptoms of schizophrenia (32). Besides, avolition might be a barrier for someone to experience pleasant events, which is also an important factor for the lack of pleasure reported by patients with schizophrenia (33). And it is plausible that patients with reduced initiation ability may have difficulty in starting the prediction of future positive events. Evidence from resting-state functional magnetic resonance image study suggests that language related brain areas such as the ventral striatum and inferior frontal gyrus are also involved in reward processing (24). While previous studies indicated that anticipatory anhedonia in schizophrenia is related to general cognitive functions and intelligence quotient (IQ), the present findings showed that it is language function rather than other cognitive factors such as immediate memory, delayed memory, attention and visuospatial skill that may influence anticipatory anhedonia. And we may speculate that language functions and anticipatory pleasure share neurobiological underpinnings partially.

As illustrated by the present study, the significantly decreased delayed memory is a risk factor for consummatory pleasure in the schizophrenia group. Other cognitive factors such as immediate memory, language, attention and visuospatial skill were not found to be correlated with consummatory pleasure. Although the present finding is quite new and the mechanisms of the associations between consummatory anhedonia and delayed memory can not be fully explained through the present research, this finding seems to be reasonable for the following reasons. On the one hand, consummatory pleasure is about in-the-monent emotional feelings during ongoing events. And it is reported that in-the-monent emotional feelings are impaired in schizophrenia patients with working memory deficits (26). On the other hand, frontal regions, which are found to be hypoactivated in anhedonia in schizophrenia (34–36), are also reported to be associated with delayed memory (37, 38). But more studies, especially longitudinal researches, are warranted to explore the explicit mechanisms. Although earlier studies reported the associations between anhedonia and cognitive functions, no study has ever revealed distinctions in specific domains of cognitive influencing factors for anticipatory and consummatory anhedonia. The present research made up for the insufficience and confirmed that anticipatory and consummatory anhedonia had different cognitive influencing factors, which is consistent with the consensus that these two dimensions of anhedonia have different features and biological mechanisms. Future works may be inspired by these findings and further investigate the detailed mechanisms underlying anticipatory and consummatory pleasure from the cognitive neuroscience perspective.

Several limitations should be noted here. First, physical anhedonia rather than social anhedonia can be measured using TEPS. Therefore, the present findings cannot be generalized to social anhedonia. Second, given the cross-sectional design of the study, the causality of cognitive impairment and anhedonia cannot be stated. Thus, further longitudinal studies are needed to elucidate the present result. Third, the heterogeneity of schizophrenia may affect the result.

In summary, we conducted a study to investigate anticipatory and consummatory anhedonia in schizophrenia and their cognitive associations. Our results showed that patients with schizophrenia exhibited reduced anticipatory pleasure and consummatory pleasure. A broad cognitive impairment was also found in patients with schizophrenia. Furthermore, language deficit is a risk factor for anticipatory anhedonia and delayed memory deficit is a risk factor for consummatory anhedonia. The results of the study have added new evidence on the influencing factors of anhedonia and provided clues for the associations between clinical manifestations of schizophrenia.

## Data Availability Statement

The raw data supporting the conclusions of this article will be made available by the authors, without undue reservation.

## Ethics Statement

The studies involving human participants were reviewed and approved by Ethics Committee of Tianjin Medical University General Hospital. The patients/participants provided their written informed consent to participate in this study.

## Author Contributions

CZ: study design. LY, XF, YC, DW, and ZW: data collection. LY and HN: statistical analysis. LY, HN, ZW, and CZ: drafting the manuscript work or revising it critically for intellectual content. LY, HN, XF, YC, DW, ZW, and CZ: approval of final version to be published and agreement to be accountable for the integrity and accuracy of all aspects of the work. All authors contributed to the article and approved the submitted version.

## Funding

This work was supported by National Key Research and Development Program of China (grant number 2018YFC1314302), National Natural Science Foundation of China (grant number 81771450), Xuhui Smart Medical Special Research Project (grant number XHZH202015), Western Medicine Guide Project of Shanghai Municipal Commission of Science and Technology (grant number 14411969000), Science and Technology Development Program of Nanjing Medical University (grant number NMUB2019107), and Medical Science and Technology Development Foundation of Nanjing Department of Health (grant number YKK20090).

## Conflict of Interest

The authors declare that the research was conducted in the absence of any commercial or financial relationships that could be construed as a potential conflict of interest.

## Publisher's Note

All claims expressed in this article are solely those of the authors and do not necessarily represent those of their affiliated organizations, or those of the publisher, the editors and the reviewers. Any product that may be evaluated in this article, or claim that may be made by its manufacturer, is not guaranteed or endorsed by the publisher.
